# Medicalization Defined in Empirical Contexts – A Scoping Review

**DOI:** 10.15171/ijhpm.2019.101

**Published:** 2019-12-21

**Authors:** Wieteke van Dijk, Marjan J. Meinders, Marit A.C. Tanke, Gert P. Westert, Patrick P.T. Jeurissen

**Affiliations:** Scientific Institute for Quality of Healthcare (IQ healthcare), Radboud Institute for Health Sciences, Radboud University Medical Center, Nijmegen, The Netherlands.

**Keywords:** Medicalization, Scoping Review, Definitions in Epirical Use

## Abstract

**Background:** Medicalization has been a topic of discussion and research for over four decades. It is a known concept to researchers from a broad range of disciplines. Medicalization appears to be a concept that speaks to all, suggesting a shared understanding of what it constitutes. However, conceptually, the definition of medicalization has evolved over time. It is unknown how the concept is applied in empirical research, therefore following research question was answered: How is medicalization defined in empirical research and how do the definitions differ from each other?

**Methods:** We performed a scoping review on the empirical research on medicalization. The 5 steps of a scoping review were followed: (1) Identifying the research question; (2) Identifying relevant studies; (3) Inclusion and exclusion criteria; (4) Charting the data; and (5) Collating, summarizing and reporting the results. The screening of 3027 papers resulted in the inclusion of 50 empirical studies in the review.

**Results:** The application of the concept of medicalization within empirical studies proved quite diverse. The used conceptual definitions could be divided into 10 categories, which differed from each other subtly though importantly. The ten categories could be placed in a framework, containing two axes. The one axe represents a continuum from value neutral definitions to value laden definitions. The other axe represents a continuum from a micro to a macro perspective on medicalization.

**Conclusion:** This review shows that empirical research on medicalization is quite heterogeneous in its definition of the concept. This reveals the richness and complexity of medicalization, once more, but also hinders the comparability of studies. Future empirical research should pay more attention to the choice made with regard to the definition of medialization and its applicability to the context of the study.

## Background

While the definition and understanding of medicalization has evolved over time, there has never been a broad consensus on its meaning.^1^ The debate started in the seventies with the notion that medicine increasingly gained social control.^2^ Zola stated that society’s explicit wish for medicine to use its controlling potential resulted in medicalization.^3^ He stated that medicine was replacing the traditional institutions that ‘shaped’ society, such as religion and law.^2^ Ultimately, this resulted in more reliance on experts.^3^ Zola criticized the assumed neutrality of this process.^3^ According to Illich, medicine gained power at the expense of people’s natural competences.^4^ This social control of medicine over people’s lives led to iatrogenic effects. Illich considered physicians’ imperialism central to this process. Although Illich never defined medicalization, he is generally understood as one of the first to address it and its consequences. Both Zola and Illich considered social control central to medicalization.

The feminist critique on medicalization centers also on social control as a central element, yet here the focus lay on medicalization resulting in professionals, traditionally men, gaining power and agency over women’s health, bodies and reproductive processes. Medical care surrounding pregnancy and delivery is an example of a well-developed field within this literature,^5,6^ but the range of topics is extensive.^7^ While medicalization is seen as inseparably gendered by some, recently attention was drawn to the medicalization of male issues, such as erectile dysfunction,^8^ soldiers war trauma^9^ and male menopause.^10^

The conceptual understanding of medicalization has shifted over time. In 1992, Conrad defined medicalization as: “*Medicalization consists of defining a problem in medical terms, using medical language to describe a problem, adopting a medical framework to understand a problem, or using a medical intervention to “treat” it* ” (p. 211).^11^ Here, Conrad placed the definitional aspect of medicalization at the core of its essence. Through the process of medicalization problems, and – if available – their solutions, come under the jurisdiction of medicine. Nonetheless, social control was not at the core of this influential definition per se.

During the past decades, a shift in the ‘engines of medicalization’ has been noticed, placing more emphasis on diverse contributors towards medicalization, such as industry and patients.^12,13^ This broader perspective served a more comprehensive understanding of medicalization. For example, it also provides the possibility to study positive effects of medicalization.^14,15^ On the other hand, Hofmann has argued that this resulted in medicalization becoming too much of an all-embracing term, and losing its critical value.^16^ He stated that medicalization has evolved over time from a critical perspective on the power-relations in medicine, to an almost all-compassing term involving all transformations in modern medicine. Furthermore, it has been argued that by focusing on the definitional issue of medicalization, the applied nature of medicine was overlooked.^1^

Parallel to the field of medicalization, adjacent research fields have developed, such as pharmaceuticalization and biomedicalization. Pharmaceuticalization is “*the process by which social, behavioural or bodily discomforts are treated, or deemed to be in need of treatment/intervention, with pharmaceuticals by doctors, patients or both* .”^17^ Biomedicalization constitutes intensified medicalization, transformed and boosted due to techno scientific innovations.^18^ Both are conscious of the corporate interests of companies, technological changes, consumerism, the influence of the media and risk.^19^ Both processes define similar mechanisms to medicalization. Therefore, it is disputed whether either constitutes a new, unique process,^18^ or in fact represent a subset of medicalization (pharmaceuticalization) or an intensified form of medicalization (biomedicalization).^20^

The literature that focused on medicalization is multifaceted and addresses many topics. Most of the work is conceptual, discussing its occurrence and essence. Empirical studies that systematically gather and analyze data are relatively rare. Such empirical studies mostly use qualitative methods, although a small sample of quantitative studies is available.^21-23^ A large share of the literature consists of ‘discussion papers’: well-informed and well-founded articles that discuss the medicalized status of a problem or situation. A non-exhaustive list of topics include the medicalization of sleep^24^; hyperactive behavior in children^25,26^; self-injuring acts^27^; and risks and genetic markers.^28,29^ Although discussion papers support the conceptual development, a major drawback is that their empirical rigorousness is uncertain. This review is among the very first to focus on the empirical translation of the concept of medicalization.

The growing body of conceptual literature on medicalization underlines the necessity of a clear understanding of its use in empirical research. How medicalization was used in empirical research has not been reviewed yet. Because the definition of the subject under study is crucial to a study, as it shapes the researchers perspective and nudges the interpretations of its results, this is a logical starting point for a review. Therefore, we categorized the various definitions used in empirical research and illustrate their similarities and differences. It is unknown whether the empirical application of medicalization is as diverse as the conceptual work, or whether the combined empirical work contributes to a shared understanding of medicalization and its mechanisms. This review aims to assist with a first step in this direction.

## Data and Methods

Given the aims of this study, a scoping review research design was adopted. Scoping reviews are characterized by the intention to ‘map’ a certain research and find what possible unanswered questions remain.^30^ We used the framework of Arksey and O’Malley for performing scoping reviews.^31^

### Identifying the Research Question

The process of a scoping review is not linear but iterative, encouraging researchers to be reflexive and repeat a step when necessary.^31^ This has proven to be very relevant to this exercise. We started with our research question: ‘What is empirically known about medicalization?’ First, all peer-reviewed research that primarily investigates medicalization was collected.

While performing these steps we discovered that studies that addressed medicalization used different definitions for a wide variety of topics. This hinders comparability. Further, we realized that many of the quantitative studies that form the core of the empirical work on medicalization, used the concept of medicalization as an interpretive frame, rather than study the essence and workings of medicalization in its actual progress. An operationalization of medicalization was not provided by any of the included studies. Thus, we were not able to test the actual construct validity of these definitions. Thus synthesizing the results of our empirical research with one meta-analysis is impossible and undesirable. Replication, comparability and robustness are difficult in any research field, but qualitative work is largely incompatible with it.^32^ Nonetheless, the results of our search exemplified that there is a robust amount of empirical research with medicalization as its subject. As said, the used definitions of the medicalization varied strongly. To further advance the concept, insight into the within-study variations were required. Empirical studies are relevant because they do apply the concept to a real life situation, indirectly testing its robustness. Therefore, the research question was iteratively adjusted to: ‘How is medicalization defined in empirical research and how do the definitions differ from each other?’

### Identifying Relevant Studies

A systematic search strategy was conducted in April 2014 in PubMed^®^, Web of Science^®^, Sociological Abstracts^®^, PsychInfo^®^, EMBASE^®^, Philosophers Index^®^, EBSCO^®^, and CINAHL^®^. The search strings were developed in cooperation with a librarian specialized in systematic reviews. References including any of the following keywords in title or abstract: “medicalization” OR “biomedicalization” OR “demedicalization” OR “biomedicalizations” OR “medicalizations” OR “demedicalizations.” Pharmaceuticalization was not one of the search words, because pharmaceuticalization is often stated to be an intensified form of medicalization, whereas biomedicalization was included as a subset of medicalization.^20^ Searches were conducted in both British and American spelling. The search string was identical for all databases. In addition, the first author (WvD) hand-searched several journals for additional results, to validate the search string. This resulted in no additional references. Duplicates, non-English references, and non-peer reviewed articles (editorials, letters, conference papers, book chapters, and dissertations) were excluded.

### Inclusion Criteria

In the first phase of the screening process, WvD screened for eligibility of the references on title and abstract. In the second phase, WvD and NdV (research assistant) screened the remaining full-texts for eligibility.

In phase one, articles that present original, empirical research with medicalization as main research topic were included. General discussions, anecdotic evidence, secondary analysis of existing data or single case studies were excluded. We chose to limit the period an article could address to the period post-World War II. We also limited the inclusion to studies conducted in high-income countries. Whether the country was a high-income country was determined with the World Bank website (accessed on February 3, 2015). Indeed, Bell and Figert argue that the emphasis within the medicalization debate lies largely on the Western context, limiting its perspective.^19^ We agree, yet we are convinced that medicalization can consist of something entirely different in the context of limited resources and little medical assistance in low income countries, compared to medicalization within affluent countries with abundant access to medical care. To improve the understanding and mechanisms of medicalization in the context of countries with few healthcare recourses was not the subject of this review. Finally, the review was restricted to peer-reviewed articles written in English.

### Charting the Data

In phase 2 the requirement of a definition of medicalization was added. Studies that failed to report how they defined medicalization were excluded. During this process, WvD and NdV met regularly for discussion. Some studies provided an overview of the medicalization debate, mentioning several definitions, but did not formally finalize the definition they would use. In such cases, we chose to retrieve definition last mentioned, because this would be what the discussion of the literature would work towards. Definition were copied literately from the studies. An overview of the data retracted in this process can be found in the supplemental material of this article.

### Collating, Summarizing and Reporting the Results

WvD and MM (the second author) studied the retrieved definitions and independently grouped them into categories. Afterwards, they met and discussed the categories. The categorization of the definitions happened on a basis of semantics, signal words and phrases. To order the categories in the resulting figure, WvD and MACT met several times to discuss the relative position the categories. At first a one-dimension ordering was pursued, but this proved incapable to capture the variation. Discussion, resulted in a second dimension to the figure.

## Results

**Figure 1:**
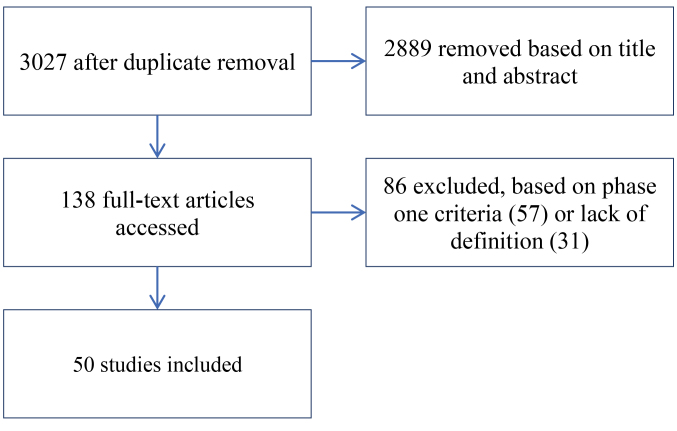
Selection Procedure of Included Studies.

The initial search resulted in 7308 potential articles of which 4281 were duplicates, resulting in 3027 unique articles. Of these 3027 article, 2977 were excluded for reasons mentioned in the methods section. 31 one empirical studies did not define medicalization. Figure 1 represents the identification and selection process.

### Allocation to a Framework Containing 2 Axes

**Figure 2:**
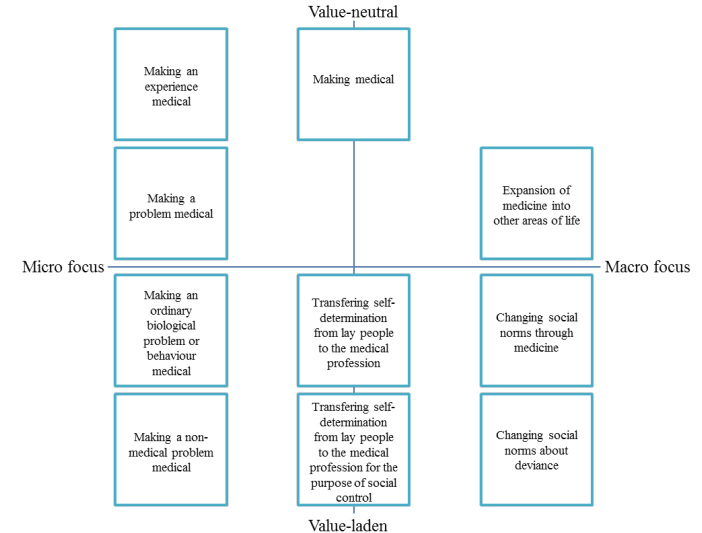
Framework of the Categories of Definitions of Medicalization Across the Axes of Micro/Macro Focus and Value-Neutral/Value-Laden.

The resulting 50 definitions were charted into ten categories (Figure 2). Most included studies quote a definition or refer to known definitions. Conrad and Zola are most often mentioned. Few are represented more than once, only Barker,^33-35^ Clarke,^36,37^ and Vainionpaa & Topo.^10,38^ Both works by Vainionpää and Topo belong to one definition category. The studies of Barker and Clarke & Lang/Clarke were allocated to different categories.

Two definitions could not be allocated, because they combined distinctive elements from across the spectrum.^39,40^ An overview of the 50 selected studies can be found in the supplemental material (Supplementary file 1, Table S1).

The ten categories were allocated in a framework containing 2 axes: one addressing the value position of the definition at stake and the other addressing its micro/macro focus. Definitions that are value-laden include a judgment of the consequences and desirability of the process of medicalization. Value-neutral definitions do not include such a judgment. Definitions with a micro focus concentrate on the individual. Definitions on the other axis concentrate on the societal implications of medicalizing a situation.

Table illustrates these ten categories. For each category, this table shows a definition from one of the included studies. For reasons of further clarification, this table provides a fictive illustration for each of these categories.

**Table:** Overview of Categories of the Definitions of Medicalization, the Articles Utilizing Those Definitions, an Example as Used in One of the Articles and an (Fictive) Illustration

**Distinctive Definition, Answering the Question ‘What Constitutes Medicalization?’**	**Studies**	**Definition Used**	**Illustration**
Making medical	^42^	“Medicalization is (ideally) a non-judgmental term, referring simply to the process of ‘making medical’*”* (Williams et al,^42^ p. 252).	All of the illustrations
Making an experience medical	^33,37,41,44-47^	“‘Medicalization,’ or the processes by which an ever wider range of human experiences come to be defined, experienced, and treated as medical conditions” (Barker,^33^ p. 21).	Signaling a rare case of feeling bloated as irritable bowel syndrome, a night of bad sleep as insomnia or normal-range shyness as social anxiety disorder
Making a problem medical	^48-50^	“Medicalization consists of defining a problem in medical terms, using medical language to describe a problem, adopting a medical framework to understand a problem, or using medical intervention to treat it” >quotes from Conrad, 1992 (Elston et al,^50^ p. 577).	Attempting to improve a negative self-image by means of cosmetic surgery
Making an ordinary biological process or behavior medical	^23, 34,51-55^	“Medicalization is the process by which formerly normal biological processes or behaviors come to be described, accepted, or treated as medical problems” (Moloney et al,^23^ p. 1429).	Approaching the aging body through a medical perspective, attempting to repair natural decline
Making a non-medical problem medical	^56-64^	“A process by which non-medical problems become defined and treated as medical problems, usually in terms of illnesses or disorders” >quotes Conrad, 2000 (Neiterman,^60^ p. 114).	Medical professionals attending people who experience loneliness and prescribing antidepressants and/or welfare arrangements
Expansion of medicine into other areas of life	^10,36,38,65-68^	[Medicalization] “refers to the ways in which medicine expands into new arenas” (Vainionpää and Topo,^38^ p. 842).	Creating calm and teachable schoolchildren by neutralizing unwanted behavior with pharmaceuticals
Changing social norms through medicine	^69-73^	“This refers to an intricate social process involving the dominance of biomedical paradigms and authoritarian models of health care in which illness experiences are understood as biological and individualistic” (Thomas-McLean,^73^ p. 630).	Change in perspective about desirability of the birth of children with severe birth defects or chromosomal defects due to availability and acceptability of prenatal testing
Changing social norms about deviance through medicine	^74,75^	“I use the term ‘medicalization’ to refer to the process by which deviant acts (*a* ) become understood to originate from a medical cause and are therefore perceived to be beyond an individual’s control; and (*b* ) are believed to be treatable through medical knowledge and the application of techniques by medical experts” (Rafalovich,^75^ p. 26).	Regarding criminal acts the result of sickness rather than badness
Transferring self-determination and decision-making from lay people to the medical profession	^76,77^	“The medical profession, on behalf of industrialism, has not only duped the public into believing that they have an effective and invaluable body of knowledge and skills but have created a dependence through the medicalization of life which has now taken away the public’s right to self-care” (Calnan,^77^ p. 561).	People changing their daily routine on doctors’ orders to meet the conditions of their complex treatment regime, for example in case of hiv-infection or Parkinson’s disease, while they felt more well and secure in their personal rhythm
Transferring self-determination and decision-making from lay people to the medical profession for the purpose of social control	^78-81^	[Medicalization is a] “process of social control whereby both deviant behavior and natural life events are reconstructed as illnesses or disorders and placed under the jurisdiction of the medical profession” (Hislop and Arber,^80^ p. 816).	Patients in long stay mental health care expected to live according to the institutional daily schedule, surrendering their privacy and autonomy to clinicians and other professionals
Not allocated	^39,40^	The definition and treatment of life problems, processes, or deviance in medical terms (Paramsee,^40^ p. 1342). Medicalization of infertility, or its treatment as a pathological condition rather than a natural or social one (Bell,^39^ p. 631).	

Neither, year of publication nor topic could be related to the categories. The 50 allocated studies were for the most part published after 2000. Several subjects are represented across the entire spectrum of the different categories, including pregnancy, children’s behavioral problems, and cosmetic surgery. Medicalization of sleep is subject to studies on the ends of both axes. Geographically the North-American continent is dominant with 19 of studies conducted in the United States and 11 in Canada (separate analysis). Several European countries are represented: the United Kingdom (10); Finland (4); Sweden (2); the Netherlands (1); France (1); and Ireland (1). One study was conducted in New Zealand. For 1 study, the country of origin of the respondents could not be determined.^41^

### Ten Categories of Medicalization

Each category is discussed with reference to Figure 2, starting with the four categories ranging from top left to bottom left. These 4 categories all have a micro perspective, but differ in the extent to which they are value-laden. The definition that is most value-neutral focuses on experiences and their medicalization. The next category concerns the medicalization of a problem: “Medicalization consists of defining a problem in medical terms, using medical language to describe a problem, adopting a medical framework to understand a problem, or using medical intervention to treat it.”^50^ This definition is quoted from Conrad.^11^ In the third of these four definitions, the definition of medicalization requires for something ordinarily biological to be present to get medicalized: “Medicalization is the process by which formerly normal biological processes or behaviors come to be described, accepted, or treated as medical problems.”^82^ Only the treatment of ordinary situations are stated to be medicalization. This makes it a less value-neutral definition than the previous category, because it makes an implicit distinction between ordinary and non-ordinary situations.

The fourth category defines medicalization as: “A process by which non-medical problems become defined and treated as medical problems, usually in terms of illnesses or disorders.”^60^ Problems that were previously not regarded as medical in nature come to be medically treated. This definition makes a distinction between medical and non-medical problems, implicating that the difference between the two groups is apparent.

For other definitions, the other end of the horizontal axis is more distinctive, focusing on the macro outcomes of medicalization. This holds for the three categories on the right site of the framework. Here, medicalization: “refers to the ways in which medicine expands into new arenas.”^38^

Other definitions go one step further, including not only the expansion of medicine into other areas of life, but also subsequently changing the social norms surrounding it: “This refers to an intricate social process involving the dominance of biomedical paradigms and authoritarian models of healthcare in which illness experiences are understood as biological and individualistic.”^73^ These definitions are more value-laden, as is represented by the other axis in the framework

The next category, in the right bottom of Figure 2, focuses on the changing norms surrounding deviance: “I use the term “medicalization” to refer to the process by which deviant acts (*a* ) become understood to originate from a medical cause and are therefore perceived to be beyond an individual’s control; and (*b* ) are believed to be treatable through medical knowledge and the application of techniques by medical experts.”^75^

The second axis concerns the values included in the definition. The remaining three categories are placed in the centre of Figure 2. The one end of this axis concerns the definitions that do not draw a (moral) judgment about the content or consequences of medicalization. When medicalization is defined as ‘making medical’ no consequence is predicted for society or the power-balance therein. Williams et al define medicalization as “(ideally) a non-judgmental term, referring simply to the process of ‘making medical.’”^83^ According to this definition, everything that belongs to the jurisdiction of medicine was once medicalized.

When medicalization is defined as “the transfer of knowledge from the lay people to the medical profession for the purpose of social control,” medicalization is perceived as an imperialist effort of the medical profession, overruling lay autonomy, representing the other end of this axis. This includes a strong power-related and value-laden consequence of medicalization as an integral aspect of the definition.

The definition that states that medicalization is the transfer of knowledge and decision-making from lay people to the medical profession is less value-laden. Calnan states: “The medical profession, on behalf of industrialism, has not only duped the public into believing that they have an effective and invaluable body of knowledge and skills but have created a dependence through the medicalization of life which has now taken away the public’s right to self-care.”^77^ Medicalization, in this definition, compromises the right of self-determination.

## Discussion

This scoping review and the resulting framework (Figure 2) provide several insights on the composition and heterogeneity of medicalization research. Firstly, the actual research topics seem not to be related to the different categories of definitions of medicalization. For example, studies about sleep were present across several categories in the spectrum, including the two ends of the value axes. This illustrates that even within the research field of medicalization, the same subject can be studied from different (conceptual) angles. It also complicates the comparability of results. Secondly, in spite of diversity in definitions, the sources that the studies based their definitions upon were dominated by one author. In 20 of the 50 studies Conrad is either quoted or referred to, as a single author or in shared authorship. While Conrad’s perspective on medicalization has evolved over the decades, his 1992 definition remains a point of reference in empirical work.^11^

These findings add up to an important discussion point. Medicalization research has a strong qualitative focus, explicating different aspects and nuances of the phenomenon. This review did not have the goal to disqualify this rich literature or to unify the perspective on the phenomenon. The goal was to map the definitions, to illustrate the diversity of the field. Differences between studies’ definitions can be entirely justifiable because of the research question or focus of the study. However, it is nonetheless relevant to notice and be transparent about them. Since none of the studies provided an operationalization of their definition, it is impossible to reflect on the construct validity of medicalization in empirical research. This is another reason to press for transparency and reflection in future research. Furthermore, our research shows that scholars chose different conceptual angles. This variety also illustrates that empirical work will always be context dependent and will highly relate on the case study at issue.

Our research resulted in a framework that can be used by scholars to classify their work and that of others. Nonetheless, a framework like ours raises new questions. For example, the framework illustrates how definitions may vary in value-ladenness. The process of medicalization results in people becoming patients. This might improve their social position and their health, but it might have a profound (possibly detrimental) effect on their life as well. Another critical remark can be made with regard to the micro/macro axis. A definition on the macro level can make it more difficult to identify the individual consequences on the micro-level. This can have important consequences for both research and policy-making. For example, if the focus lies solely on the negative macro consequences of a newly medicalized situation, for example its costs, individual benefits can easily be overlooked. Avoiding this problem by choosing the most neutral definition, ‘the process of making medical,’ seems to address this problem. Yet, this definition is possibly too general to be of empirical use. This reveals a trade-off between specific and general definitions of medicalization. Further, as mentioned in the introduction, the conceptual definition of medicalization is crucial to a study, because it frames the perspective of the research team. What a researcher perceives as essential to medicalization, influences his or her perspective on the interpretation of the results. For example, when the influence and power of the medical profession is stated to be essential for the definition of medicalization, this probably influences the interpretation of the results. Subsequently, their understanding of health and healthcare are likely to differ from studies that use definitions focus on other elements.

## Limitations

A limitation of this study is that the review process was guided by the empirical studies that were identified. It is not possible to conclude that every conceptual definition of medicalization has been applied. Furthermore, we did not address whether the chosen definition was the most valid one per study. It would require an in-depth analysis of each study to draw conclusions about the application of the used definition to the studies contexts and results. The sample of 50 studies was too big to perform these analyses. Therefore, we are unable to verify whether the methods and results per study agree with the allocation of the study in the framework. This makes it impossible to state anything about the empirical applicability of the definitions. Future research should pay attention to these difficulties and should be more responsive and reflective about the choices made within each study, also with regard to the chosen definition and its applicability to the research context.

With regard to the scoping exercise, we found 31 studies with medicalization as its subject that failed to define medicalization. We did not distillate the implicit definition from these studies, because we found this too sensitive for misinterpretation. Further, the grouping of the definitions and the charting of the figure were an interpretative exercise. This is both a strength and a possible weakness of this method. To minimize the risk, we discussed allocation of the definitions and the categories to the figure regularly within our project group. This resulted in a figure that all authors regarded as robust and representative for the included conceptual definitions.

## Implications for Future Research

This scoping review showed that empirical research about medicalization has a broad scope. This portrays the richness and variety of the field. Nonetheless, we reveal that the understanding of what medicalization constitutes of differs as much within empirical studies as it does in the conceptual literature. To advance the understanding of the mechanisms of medicalization, future research should be attentive to these differences, defining their study subject accurately, to enable the further development of the concept and to connect the conceptual and the empirical literature.

## Acknowledgements

We thank Nynke de Vries for her assistance in the review process.

## Ethical issues

Not applicable.

## Competing interests

Authors declare that they have no competing interests.

## Authors’ contributions

WvD, MJM, MACT, GPW, and PPTJ conceived and designed the review process and analysis. WvD and MJM collected the data. WvD and MJM performed the data analysis. All authors discussed the results and contributed to the final manuscript.

## Supplementary files


Supplementary file 1 contains Table S1.
