# Supplementary material for: Medicalization Defined in Empirical Contexts – A Scoping Review
Source: Int J Health Policy Manag. 2019 Dec 21;9(8):327–34. doi: 10.15171/ijhpm.2019.101 (PMC7500387; doi:10.15171/ijhpm.2019.101)
Supplement: Supplementary file 1 — contains Table S1. [file ijhpm-9-327-s001.pdf]

Supplementary file 1

Table S1. Overview of Included Studies

Authors	Year	Title	Journal	Primary subject of study	How is medicalization defined?	Topic	Country of origin
Adams	2013	Medicalization and the Market Economy: Constructing Cosmetic Surgery as Consumable Health Care	Sociological Spectrum: Mid-South Sociological Association	This research is designed to examine how individuals frame their decisions to undergo cosmetic surgery in economic terms	Medicalization of the body, whereby reasonably normal appearances are problematised, yet can be remedied through medical intervention > Refers to Dull and West, 1997; Sullivan, 2001	Cosmetic surgery	United States
Arney & Rafalovich	2007	Incomplete syllogisms as techniques of medicalization: the case of direct-to-consumer advertising in popular magazines, 1997 to 2003	Qualitative Health Research	How does advertising invite the reader to explore her or his own experiences within the context of a particular mental disorder? How does advertising define individual deviance as medical and encourage the seeking of medical attention?	Defining a problem in medical terms, using a medical language to describe a problem, adopting a medical framework to understand a problem, or using a medical intervention to 'treat' it > quotes from Conrad & Schneider, 1980, p.211	Antidepressant medication	United States
Barker	2008	Electronic support groups, patient-consumers, and medicalization: the case of contested illness	Journal of health and social behavior	What role do electronic support groups play in the process of consumer-driven medicalization?	"Medicalization," or the processes by which an ever wider range of human experiences come to be defined, experienced, and treated as medical conditions	Fibromyalgia	World Wide Web (in English)
Barker	2011	Listening to Lyrica: contested illnesses and pharmaceutical determinism	Social Science & Medicine	I describe the role pharmaceutical companies and pharmaceuticals play in	Medicalization is the process by which ever more aspects of the human condition are defined	Fibromyalgia	World Wide Web (in English)

				promoting and legitimating contested diagnoses	and treated as medical in character		
Barker	2014	Mindfulness meditation: Do-it-yourself medicalization of every moment	Social Science & Medicine	Both selfhelp and alternative healing approaches have been identified as encouraging as well as resisting medicalization. I address this contradiction using the case of mindfulness	Defining a problem in medical terms, usually as an illness or disorder, or using a medical intervention to treat it > quotes from Conrad, 2005, p.3	Mindfulness	International, focus on US (Amazon.com, data in English language)
Becker & Nachtigal	1992	Eager for medicalization: the social production of infertility as a disease	Sociology of Health & Illness	The social and cultural basis of medicalization is explored through an examination of infertility, a social condition that has recently been recast as a disease	Medicalization refers to the process by which human experiences are redefined as medical problems	Infertility	United States
Bell	2010	Beyond (financial) accessibility: inequalities within the medicalization of infertility	Sociology of Health & Illness	I examine the process of medicalization and how it contributes to the development of disparities through its perpetuation of dominant ideologies	Medicalization of infertility, or its treatment as a pathological condition rather than a natural or social one	Infertility	United States
Binney, Estes & Ingman	1990	Medicalization, public policy and the elderly: Social services in jeopardy?	Social Science & Medicine	This paper examines the medicalization of community-based services for the elderly; a process of restructuring to provide more highly medical services to a frail older population at the expense of providing a broader range of social and supportive services to older persons with varying levels of need	The term medicalization is used to refer to the substitution of medical care, including medical services, for care which was formerly nonmedical, or the substitution of all or part of a medical model of care for what was formerly a nonmedical model > refers to Swan, 1985	Community-based services for the elderly	United States

Boero	2007	All the News that's Fat to Print: The American "Obesity Epidemic" and the Media	Qualitative Sociology	I explore the process by which the “obesity epidemic” has come to be defined as a social problem at the same time as it is framed as a problem of individuals.	The moral model of fatness shifted to a medical model in which “obesity” was designated as a disease to be treated through medical intervention > refers to Sobal, 1995	Obesity	United States
Bransen	1992	Has Menstruation Been Medicalised? Or Will It Never Happen	Sociology of Health & Illness	In what terms do women talk about menstruation and about menstrual-cycle-related problems or illness? And how do these genres frame the relationship between medical expert and layperson?	The rendering of life experiences as processes of health disorders, which can be discussed in medical terms only and to which only medical solutions can be applied > refers to Baart & Baerveldt, 1986	Menstruation	?
Brubaker	2007	Denied, embracing, and resisting medicalization: African American teen mothers' perceptions of formal pregnancy and childbirth care	Gender & Society	How does the stigma of being a pregnant African American teen shape the decisions and behaviours of these teens regarding medical care and their responses to such care?	The process by which behaviours or conditions take on medical meanings, "that is, defined in terms of health and illness". It is a process in which "medical practice becomes a vehicle for eliminating or controlling problematic experiences that are defined as deviant, for the purpose of securing adherence to social norms" > quotes from Riesmann, 1983, p.4	Teen pregnancy	United States
Calnan	1984	Women and medicalization: an empirical examination of the extent of women's dependence on medical	Social Science & Medicine	Which method do women prefer to detect breast cancer: self-examination (less medicalised) or mammography (medicalised option)?	The medical profession, on behalf of industrialism, has not only duped the public into believing that they have an effective and invaluable body of	Breast cancer screening	United Kingdom (England)

		technology in the early detection of breast cancer			knowledge and skills but have created a dependence through the medicalization of life which has now taken away the public's right to self-care > refers to Illich, 1975		
Chang & Christakis	2002	Medical Modelling of Obesity: A Transition from Action to Experience in a 20th Century American Medical Textbook	Sociology of Health & Illness	Our aim is to (...) conduct an in-depth investigation of how its conceptualisation of obesity, a presumably unambiguous and cohesive object of knowledge, can undergo considerable transformation	Medicalization refers to the process by which certain behaviours or conditions are defined as medical problems, and medical intervention becomes the focus of remedy and social control > refers to Reissman, 1983; Fox, 1988; Conrad & Schneider, 1992	Obesity	United States
Clarke & Lang	2012	Mothers Whose Children Have ADD/ADHD Discuss Their Children's Medication Use: An Investigation of Blogs	Social work in Health care	This research set out to examine how mothers describe what they consider to be the responsibilities and duties of mothering a child with ADD/ADHD in conversations with one another on the internet	Bio-medical perspective predominates over what might otherwise have been viewed as moral, religious, legal, community, or other sorts of issues > refers to Conrad, 2005	Medication use by children with ADHD/ADD	World Wide Web (in English)
Clarke	2013	Medicalization and changes in advice to mothers about children's mental health issues 1970 to 1990 as compared to 1991 to 2010: evidence from Chatelaine magazine	Health, Risk & Society	I aim to contribute to our understanding of the ways in which women's magazines contribute to popular understanding of children's mental health issues	Medicalization can be defined as an expansion in the aspects of life considered to be of relevance to medical care	Children's mental health issues	Canada
Coveney, Nerlich & Martin	2009	Modafinil in the media: Metaphors, medicalization and the body	Social Science & Medicine	How is modafinil discursively constructed in the British print media? How does this influence	[Medicalization] is a bi-directional and multi-faceted process through which human	Sleep	United Kingdom

				the configuration and reconfiguration of the body in popular consciousness? How and where is 'medicalization' deployed? And to what effect? What does this tell us more generally about cultural attitudes towards human enhancement?	differences are transformed into pathologies, diagnosable disorders and treatable conditions > refers to Conrad, 1992		
Elston et al.	2002	Violence against Doctors: A Medical(ised) Problem? The Case of National Health Service General Practitioners	Sociology of Health & Illness	Is violence towards clinicians (GPs) medicalised? To aswer this question, there are two parts to the empirical analysis: an examination of the framing of violence against GPs as a policy issue; followed by analysis of individual GPs' response to violent incidents.	Medicalization consists of defining a problem in medical terms, using medical language to describe a problem, adopting a medical framework to understand a problem, or using medical intervention to treat it > quotes from Conrad, 1992, p.211	Violence towards clinicians	United Kingdom
Fainzang	2013	The other side of medicalization: Self-medicalization and self-medication	Culture, Medicine & Psychiatry	I demonstrate that while self-medication often results from the reproduction and renewal of a previous medical opinion, it may also result from a personal decision to suggest a medical interpretation for a problem and therefore to resort to medical treatment	Medicalization thus designates the extension of medical jurisdiction into the social lives of individuals and is perceived as the medical management of a phenomenon that might have been—or which previously was—managed differently > refers to Zola, 1992	Self-medicalization	France
Gammell & Stoppard	1999	Women's experiences of treatment of depression: Medicalization or empowerment?	Canadian Psychology	We investigate women's experiences in relation to being diagnosed with and treated for depression, and how participants came to be diagnosed as	When a woman's distress is conceptualised as a medical problem, one for which a drug (such as an anti-depressant) is prescribed by a physician, her	Depression in women	Canada

				depressed, their understandings of what caused their depression, and their experiences of the treatment they received	problems become "medicalised" (defined as a medical problem)		
Harvey	2013	Medicalization, pharmaceutical promotion and the Internet: a critical multimodal discourse analysis of hair loss websites	Social Semiotics	How is male pattern baldness framed and medicalised by pharmaceutical websites advertising for a particular solution?	Medicalization is the socio-cultural process whereby the ordinary processes of life become ‘‘defined and treated as medical problems, usually in terms of illnesses or disorders’’ > quotes from Conrad, 1992, p.209	Male pattern baldness	World Wide Web (in English)
Hislop & Arber	2003	Understanding women's sleep management: beyond medicalization-healthicization?	Sociology of Health & Illness	We explore the extent to which the concepts of medicalization and healthicization provide appropriate models for understanding the management of women’s sleep disruption	[Medicalization is a] process of social control whereby both deviant behaviour and natural life events are reconstructed as illnesses or disorders and placed under the jurisdiction of the medical profession > refers to Conrad, 1992	Sleep problems in women	United Kingdom (England)
Hogle	2001	Chemoprevention for Healthy Women: Harbinger of Things to Come?	Health (UK)	How do women respond to advertising messages suggesting that they may be something other than ‘normal,’ and that they should use a chemical technology because of this status?	Through processes of medicalization, an increasing variety of physical and behavioural conditions are seen as disorders needing biomedical intervention	Chemoprevention for women at risk of breast cancer	United States
Holmqvist	2009	Medicalization of unemployment: Individualizing social issues	Work, employment & society	This article examines a phenomenon that can be called the ‘medicalization of unemployment’	The process by which human behaviours become defined and treated as medical problems	Unemployment	Sweden

		as personal problems in the Swedish welfare state			and issues > refers to Ballard & Elston, 2005; Schram, 2000		
Hyde et al.	2006	Social regulation, medicalization and the nurse's role: insights from an analysis of nursing documentation	International Journal of Nursing studies	We elucidate how the Roper–Logan–Tierney (RLT) model of nursing gives formal recognition to the medicalization of ordinary daily activities, and creates a framework for nurses through which the process of medicalization is facilitated	More and more realms of daily life have come to be related to 'health' or 'illness > refers to Zola, 1992; 1984	Nursing	Ireland
Jacob, Gagnon & McCabe	2014	From distress to illness: a critical analysis of medicalization and its effects in clinical practice	Journal of Pshycatric & Mental Health Nursing	This study examines the particularities of lipodystrophy in relation to the female body and how this condition affects the lives of HIV-positive women by reconfiguring their body in unexpected ways	When a specific aspect of the body becomes the focus of medical attention, there is a process by which it is claimed, controlled, and brought into medical ideology > quotes from Mason & Mercer, 1999, p.57	Effects of antiretroviral therapy on HIV-positive women	Canada
Kilty	2012	'It's like they don't want you to get better': Psy control of women in the carceral context	Feminism & Psychology	This article examines how women incarcerated in provincial and federal prisons in Canada experience medicalization as the predominant form of correctional psy intervention	Medicalization is a process through which 'an entity' that it is not 'ipso facto a medical problem', is responded to as a kind of illness > quotes from Conrad, 2007, p.5-6	Physical treatment of incarcerated women	Canada
Lee, Macvarish & Sheldon	2014	Assessing child welfare under the Human Fertilisation and Embryology Act 2008: a case study in medicalization?	Sociology of Health & Illness	The aim of the study was to find out how this change to the law had impacted on practice. In describing what we found, we also make a contribution to scholarship about the medicalization of reproduction	The process through which non-medical problems become defined and treated as medical problems > quotes from Conrad, 2007, p.4	Welfare of the child assessments pre-conception before infertility treatment	United Kingdom

Malacrida	2004	Medicalization, ambivalence and social control: mothers' descriptions of educators and ADD/ADHD	Health: An Interdisciplinary Journal for the Social Study of Health, Illness & Medicine	How do mothers understand the role of educators in the medicalization of their children's behaviour?	Medicalization is a definitional problem, the process whereby non-medical problems become routinely understood and handled as illnesses or disorders rests on the ability of medicine to name and define the problem in medical language, to construct the individuals who present the problem in medical terms like ‘patient’ or ‘sufferer’ and to organise the ideal response to the problem along lines of medical treatment and intervention > refers to Conrad, 1992	Perceptions of the teachers role in ADHD/ADD diagnosis of children	Canada & United Kingdom
McLeod et al.	2004	Public attitudes toward the use of psychiatric medications for children	Journal of health and social behavior	We analyse nationally representative data on the public’s willingness to give psychiatric medications to children and the social correlates of that willingness	Children’s emotional and behavioural problems have become medicalised—defined and treated as medical problems and deferred to the supervision of the medical profession	Medication use by children with ADHD/ADD	United States
Melick, Steadman & Cocozza	1979	The medicalization of criminal behavior among mental patients	Journal of health and social behavior	Through a comparison of the arrest rates of mental patients released during 1968 and 1975 with the general population rates, the relevance of this process to criminal behaviour is examined	This process of defining deviant behaviour as illness and mandating or licensing physicians to treat it > refers to Conrad, 1975	Arrest rates of mental patients	United States

Merianos, Vidourek & King	2013	Medicalization of Female Beauty: A Content Analysis of Cosmetic Procedures	Qualitative Report	The purpose of this study is to conduct a content analysis of brochures made available to customers from cosmetic surgery centers located in one metropolitan area and to examine what if any framing techniques are used to encourage females to undergo cosmetic procedures	Medicalization is a process that can be defined in which nonmedical problems are describes in terms of medical problems such as illnesses and disorders > refers to Conrad, 1992	Cosmetic surgery	United States
Moloney, Konrad & Zimmer	2011	The Medicalization of Sleeplessness: A Public Health Concern	American Journal of Public Health	We explored the idea that the US epidemic of insomnia may be, in part, facilitated by medicalization	Medicalization is the process by which formerly normal biological processes or behaviours come to be described, accepted, or treated as medical problems > Refers to Conrad & Schneider, 1992	Sleeplessness	United States
Moreira	2006	Sleep, health and the dynamics of biomedicine	Social Science & Medicine	How is sleep and health related from a sociological perspective?	The changes in the understanding of the relationship between sleep and health are the outcome of a negotiated expansion of the medical boundaries of knowledge and treatment. The medicalization perspective emphasises the control and constraints on action derived from medical knowledge > refers to Williams, 2002	Sleep	World Wide Web (in English)
Neiterman	2013	Sharing bodies: The impact of the biomedical model of	Healthcare Policy	This paper explores how women experience their transition to	A process by which nonmedical problems become defined and	Pregnancy	Canada

		pregnancy on women's embodied experiences of the transition to motherhood		motherhood as a process of embodiment that is shaped by biomedical culture	treated as medical problems, usually in terms of illnesses or disorders > Quotes from Conrad, 2000, p.324		
Norris et al.	2011	Medicalization or under-treatment? Psychotropic medication use by elderly people in New Zealand	Health Sociology Review	Our research aimed to investigate whether patterns of prescribing matched patterns of need for medications	The social processes by which non-pathological problems come to be understood and treated as medical conditions > refers to Conrad 2005; 2007; Conrad & Leiter, 2004	Psychotropic medication use amongst older people	New Zealand
Oinas	1998	Medicalization by Whom? Accounts of Menstruation Conveyed by Young Women and Medical Experts in Medical Advisory Columns	Sociology of Health & Illness	This paper addresses knowledge claims about the body: whose knowledge matters when discussing menstruation between young women and medical professionals?	With this concept sociologists have tried to capture the transfer of knowledge and decision-making concerning health from lay people to the medical profession > refers to Zola, 1972; Conrad & Schneider, 1980; Conrad, 1992	Menstruation	Finland
Padamsee	2011	The pharmaceutical corporation and the 'good work' of managing women's bodies	Social Science & Medicine	How does the pharmaceutical industry (try to) affect the care of women in a company produced magazine about gynaecological health and care?	Medicalization: the definition and treatment of life problems, processes, or deviance in medical terms > refers to Zola, 1972	Pharmaceutical industry communication towards physicians	International publication of pharmaceutical publication
Parry	2008	We wanted a birth experience, not a medical experience: exploring Canadian women's use of midwifery	Health care for women international	My purpose was to explore women's choice of midwifery, including their perceptions and experiences with medicalization	Biomedical tendency to pathologise otherwise normal bodily processes and states. Such pathologisation leads to incumbent medical management > quotes Inhorn, 2006, p.354	Pregnancy and midwifery	Canada

Polonijo & Carpiano	2008	Representations of Cosmetic Surgery and Emotional Health in Women's Magazines in Canada	Women's Health Issues	The present study takes a closer look at how features of emotional health are constructed in relation to cosmetic surgery in popular women's magazines in Canada	Medicalization occurs when seemingly nonmedical problems become defined as medical issues > refers to Conrad, 2005	Cosmetic surgery representation in women's magazines	Canada
Rafalovich	2005	Relational Troubles and Semiofficial Suspicion: Educators and the Medicalization of "Unruly" Children	Symbolic interaction	This study details how educators conceptualise the "nature" of ADHD children, including how they frame such children's abilities and disabilities	I use the term "medicalization" to refer to the process by which deviant acts (a) become understood to originate from a medical cause and are therefore perceived to be beyond an individual's control; and (b) are believed to be treatable through medical knowledge and the application of techniques by medical experts	Children's behavioural problems and ADHD diagnosis	Canada & United States
Schierenbeck	2010	Medicalization of sickness absence	Work	This article explores variations in absence from work due to sickness as a result of medicalization	The process by which previously defined nonmedical problems become defined and treated as medical problems	Sickness absence	Sweden
Selin	2011	Implementation of substitution treatment in Finland: Beyond rationalisation and medicalization	NAT Nordisk alkohol & narkotikatidskrift	Finnish treatment of drug abuse has during the last two decades shifted from a predominantly psychosocial approach to a more medical mode. My aim is to show that labelling this development as 'medicalization' or 'rationalisation' as a form of medical progress will not	Which redefines social problems as medical problems > refers to Gomart & Hennion, 1999; Murto, 2002	Drug abuse	Finland

				increase our understanding of the change.			
Thomas-McLean	2004	Memories of treatment: the immediacy of breast cancer	Qualitative Health Research	What are women's experiences of embodiment after breast cancer?	This refers to an intricate social process involving the dominance of biomedical paradigms and authoritarian models of health care in which illness experiences are understood as biological and individualistic > refers to Walters, 1994	Embodiment after breast cancer	Canada
Torres	2014	Medicalizing to demedicalize: Lactation consultants and the (de) medicalization of breastfeeding	Social Science & Medicine	This paper uses the domain of breastfeeding in the U.S. and the work of International Board Certified Lactation Consultants to refine the concept of medicalization and demedicalization	A process by which nonmedical problems become defined and treated as medical problems, usually in terms of illness and disorders > quotes from Conrad, 2007, p.4	Breastfeeding and lactation consultants	United States
Vainionpaa & Topo	2005	The making of an ageing disease: the representation of the male menopause in Finnish medical literature	Aging & Society	The aim of this article is to study the presentation of the male menopause in Finnish medical teaching and professional literature	[Medicalization] refers to the ways in which medicine expands into new arenas > Zola, 1972	Male menopause	Finland
Vainionpaa & Topo	2006	The construction of male menopause in Finnish popular magazines	Critical Public Health	In this study we investigated the construction of male menopause and related hormonal treatments in Finland from the point of view of the medicalization of ageing	By medicalization we refer to the ways in which medicine expands to new arenas that were previously not defined to be part of the field of medicine > refers to Zola, 1972	Male menopause	Finland
Van Brummen & Griffiths	2013	Working in a medicalised world: the experiences of	International Journal of Palliative Nursing	This study explored the nature and experience of caring for	Illich identified that modern society has turned personal	Birth and death experiences from	United Kingdom

		palliative care nurse specialists and midwives		those at both ends of life's continuum: birth and death. In particular the practices that have evolved to counter the medicalization of care	challenges into technical problems requiring medical treatment > refers to Illich, 1974	the perspectives of midwives and palliative care clinical nurse specialists	
Venn, Meadows & Arber	2013	Gender differences in approaches to self-management of poor sleep in later life	Social Science & Medicine	We seek to understand the influence of gender on the different approaches to managing poor sleep by older men and women through the conceptual framework of existing theoretical debates on medicalization, healthicization and 'personalization'	Medicalization occurs when previously non-medical problems are defined and treated as medical problems, usually in terms of illnesses or disorders, or when a medical intervention is used to treat the problem > quotes from Conrad & Leiter, 2004, p. 825	Managing poor sleep	United Kingdom (England)
Westfall & Benoit	2004	The rhetoric of "natural" in natural childbirth: childbearing women's perspectives on prolonged pregnancy and induction of labour	Social Science & Medicine	This article aims to discover birthing women's own views on prolonged pregnancy, whether they believe some kind of intervention is warranted, and, if so, when and what kind of intervention is desirable	The process whereby increasingly more aspects of everyday life fall under medical influence and control > refers to Zola, 1983	Prolonged pregnancy	Canada
Williams et al.	2008	Medicalization and beyond: the social construction of insomnia and snoring in the news	Health: An Interdisciplinary Journal for the Social Study of Health, Illness & Medicine	This article contributes to sociological debates on sleep, the media and medicalization through a critical exploration and examination of the social construction of two common sleep problems, insomnia and snoring	Medicalization is (ideally) a non-judgemental term, referring simply to the process of 'making medical'	Sleep problems (insomnia and snoring)	United Kingdom
